# Using Digital Health Technologies to Understand the Association Between Movement Behaviors and Interstitial Glucose: Exploratory Analysis

**DOI:** 10.2196/mhealth.9471

**Published:** 2018-05-03

**Authors:** Andrew P Kingsnorth, Maxine E Whelan, James P Sanders, Lauren B Sherar, Dale W Esliger

**Affiliations:** ^1^ National Centre for Sport and Exercise Medicine School of Sport, Exercise and Health Sciences Loughborough University Loughborough United Kingdom; ^2^ Leicester Biomedical Research Centre Leicester General Hospital Leicester United Kingdom

**Keywords:** accelerometry, glucose, physical activity, physiological monitoring, sedentary time

## Abstract

**Background:**

Acute reductions in postprandial glucose excursions because of movement behaviors have been demonstrated in experimental studies but less so in free-living settings.

**Objective:**

The objective of this study was to explore the nature of the acute stimulus-response model between accelerometer-assessed physical activity, sedentary time, and glucose variability over 13 days in nondiabetic adults.

**Methods:**

This study measured physical activity, sedentary time, and interstitial glucose continuously over 13 days in 29 participants (mean age in years: 44.9 [SD 9.1]; female: 59%, 17/29; white: 90%, 26/29; mean body mass index: 25.3 [SD 4.1]) as part of the Sensing Interstitial Glucose to Nudge Active Lifestyles (SIGNAL) research program. Daily minutes spent sedentary, in light activity, and moderate to vigorous physical activity were associated with daily mean glucose, SD of glucose, and mean amplitude of glycemic excursions (MAGE) using generalized estimating equations.

**Results:**

After adjustment for covariates, sedentary time in minutes was positively associated with a higher daily mean glucose (mmol/L; beta=0.0007; 95% CI 0.00030-0.00103; *P*<.001), SD of glucose (mmol/L; beta=0.0006; 95% CI 0.00037-0.00081; *P*<.001), and MAGE (mmol/L; beta=0.002; 95% CI 0.00131-0.00273; *P*<.001) for those of a lower fitness. Additionally, light activity was inversely associated with mean glucose (mmol/L; beta=−0.0004; 95% CI −0.00078 to −0.00006; *P*=.02), SD of glucose (mmol/L; beta=−0.0006; 95% CI −0.00085 to −0.00039; *P*<.001), and MAGE (mmol/L; beta=−0.002; 95% CI −0.00285 to −0.00146; *P*<.001) for those of a lower fitness. Moderate to vigorous physical activity was only inversely associated with mean glucose (mmol/L; beta=−0.002; 95% CI −0.00250 to −0.00058; *P*=.002).

**Conclusions:**

Evidence of an acute stimulus-response model was observed between sedentary time, physical activity, and glucose variability in low fitness individuals, with sedentary time and light activity conferring the most consistent changes in glucose variability. Further work is required to investigate the coupling of movement behaviors and glucose responses in larger samples and whether providing these rich data sources as feedback could induce lifestyle behavior change.

## Introduction

### Background

Despite the well-documented health benefits of physical activity (PA) for the prevention of chronic diseases [1,2], accelerometry data from nationally representative samples suggest that only a small percentage of individuals are sufficiently active [3-5]. The UK PA guidelines propose that regular PA can reduce the risk of developing chronic diseases such as type 2 diabetes and coronary heart disease [6]. However, this requires individuals to undertake PA *now* to receive their health return on investment *later* in life. In psychology, the term temporal discounting describes how the value of a reward decreases as the delay to attainment increases [7]. Consequently, when the *reward* (eg, decreased morbidity and mortality risk) occurs too far into the future, the immediate costs (ie, effort to be active) outweigh the future benefits, and individuals are unlikely to carry out preventative measures such as engaging in more PA to avoid lifestyle-related chronic disease [8].

One potential solution to this is to show individuals the physiological consequences of their PA (or lack thereof) in a more immediate fashion. Acute physiological changes have been demonstrated by undertaking PA, such as reduced postprandial responses by completing 2 min of light (walking at 3.2 km/h) and moderate intensity (5.8-6.4 km/h) activity breaks every 20 min over a 5-hour period. Compared with uninterrupted sitting, both activity conditions lowered the net glucose response to a standardized drink (light: −1.7 mmol/L; moderate: −2.0 mmol/L) [9]. Similarly, significant attenuations of postprandial glucose were also observed in a large trial of normal weight adults when regular activity breaks were undertaken (−866.7 IU/L·9 h) [10], in postmenopausal women [11], and in office workers when asked to stand for 4 hours (43% lower excursion to a standardized lunch) [12]. The emergence of wearable technologies now allows many people to track their own behaviors and increasingly, their own health, in impressive detail. Given that most wearable technologies measure the volumetric dose of PA as standard, consumers are increasingly seeking more comprehensive information on how their actions are influencing their health [13], and therefore, quantifying the effect of movement behaviors upon acute health outcomes is an exciting development.

Although studies exist that examine the relationship between glucose and PA in a free-living setting, most involve a laboratory component or conditions that stipulate participants conduct a set program of behaviors [14-16]. This lacks some generalizability given that individuals’ lives are often not as regimented as a research protocol, and the introduction of between and within participant variation is key to determine if findings are robust. One study investigating behavior and glucose responses in individuals with type 2 diabetes outside the laboratory demonstrated that time spent in hyperglycemia was positively associated with sedentary time [17]. If these physiological consequences of small movement choices can be represented as personalized feedback (ie, glucose concentrations), it may help support individuals to be more physically active or reduce time spent sedentary [18].

### Study Aim

There are considerable gaps in our understanding of the acute physiological changes that PA and sedentary time can have upon glucose within free-living settings. Therefore, the aim of this study was to determine if there was a relationship between accelerometer measured PA, sedentary time, and measures of glucose variability over 13 days using glucose monitoring in nondiabetic adults.

## Methods

### Sample

Data used for this study were collected as part of the Sensing Interstitial Glucose to Nudge Active Lifestyles (SIGNAL) research program which aims to use PA, sedentary time, and glucose data to investigate the physiological consequences of movement. The study took place at the National Centre for Sports and Exercise Medicine at Loughborough University from May 2016 to September 2016. All participants gave their written informed consent, and the study was approved by the Loughborough University Human Participants Ethical Sub-Committee (R15-P142).

Study inclusion criteria required participants to be in the age range of 30 to 60 years and not have a current clinical diagnosis of diabetes (type 1 or type 2). Participants that had fasting glucose concentrations above the prediabetic threshold of ≥5.6 mmol/L were able to participate within the study. Exclusion criteria included taking diabetes medication, being pregnant, having any mobility-related musculoskeletal problems, or undertaking any structured exercise training.

### Study Design

Participants attended a 2-hour morning appointment (AM only) and were asked to adhere to the following pretesting guidelines before their appointment: refrain from food or drink (except water) for a minimum of 8 hours before, drink a glass of water at least 1 hour before, and refrain from any strenuous activity 24 hours before.

### Study Measurements

After arrival, informed consent was taken, and then, a Physical Activity Readiness Questionnaire was completed to ensure participant safety [19]. Any positive answers were dealt with by a clinically trained member of the study team. Once cleared for participation, a seated blood pressure reading (Omron 705IT, Omron, United Kingdom) and a fasting capillary blood test were undertaken. Two finger prick blood samples were collected and analyzed using point-of-care devices for total cholesterol, high-density lipoprotein cholesterol, low-density lipoprotein cholesterol, triglycerides and glucose (Lipid Profile Glucose Cartridge, Cholestech LDX Analyzer, Alere, Massachusetts, United States), and glycated hemoglobin (Afinion HbA1c, Afinion Analyzer, Alere, Massachusetts, United States). Height, weight, body composition, and waist circumference were measured once using a stadiometer (SECA 213, SECA, Germany), bioelectrical impedance scale (Tanita MC780MA, Tanita, The Netherlands), and tape measure (HaB International Ltd, United Kingdom), respectively. Waist circumference was measured using the average of two measurements, with a third being conducted if the first two exceeded 3 cm.

It has been demonstrated that the glucose response to PA can be influenced by cardiorespiratory fitness [20]; therefore, fitness assessments in the form of combined left and right hand grip scores (Takeii analogue dynamometer, Takei Scientific Instruments Co, LTD, Japan), quadriceps maximal voluntary contraction (G200 Knee Extension, DAVID Health Solution Ltd., Finland), and sub maximal fitness (modified Canadian Aerobic Fitness Test, mCAFT [21]) were conducted. After completion of all study profiling measurements, participants were given an accelerometer and glucose monitoring device to wear for 14 days (13 of which were complete days).

### Accelerometry

An ActiGraph accelerometer (wGT3X-BT Monitor, ActiGraph, Pensacola, United States) was fitted around the waist over the right hip (midclavicular line) of each participant. The activity monitor was worn during waking hours only (ie, removed for sleeping) and was only removed if participants engaged in water-based activities (eg, swimming or bathing). All devices were initialized 1 hour into the appointment (day 1) and were given a stop time of midnight on the last day of wear (day 14) to account for any potential issues in deployment. Measurement frequency was set to 100 Hz, and devices were downloaded into 60 second epoch files using ActiLife (ActiGraph) version 6.13.2. Files were processed using KineSoft (KineSoft) version 3.3.80.

Nonwear was defined as 60 seconds of consecutive zeros with allowance of 2 minutes of nonzero interruptions, and a valid day was defined as ≥600 minutes of valid monitor wear [4]. Counts per minute (CPM) cut-points for vertical axis data were used to define sedentary time (0-99 CPM), light physical activity (100-2019 CPM), and moderate to vigorous physical activity (MVPA; ≥2020 CPM) [4].

### Glucose Monitoring

A flash glucose monitor (Freestyle Libre, Abbott Laboratories, Illinois, United States), hereon referred to as a glucose monitor, was used to measure glucose concentrations over 14 days. The sensor is attached to the arm via an adhesive patch, and a handheld reader device downloads data from the sensor via near field communication. Interstitial glucose concentrations were captured by the sensor every 15 min and when users scanned the sensor using the handheld device. Other devices require frequent calibration using a capillary blood sample every 4 to 12 hours; however, the Freestyle Libre is factory calibrated and does not require any finger pricks during wear without significant loss of accuracy [22]. It has also been shown to be accurate in individuals with type 1 and type 2 diabetes against capillary blood measurements, and readings are not affected by body mass index (BMI) or age [23].

After deployment, a Tegaderm patch (3M, Minnesota, United States) was applied over the sensor to encourage a firm attachment. Additional patches were provided to participants in the event that patches became dirty or peeled off. Manufacturer guidelines specify that the sensor be scanned at least once every 8-hour period to avoid data loss. In this study, participants were asked to scan at least once every 7 hours, and they could also see their glucose concentrations in real time in an effort to minimize data loss. Missing data were anticipated as participants may sleep over 8 hours; therefore, participants were encouraged to scan before going to sleep and upon waking. If the sensor was removed prematurely (ie, because of an adhesion issue or a sensor error) during the first few days, redeployment took place. However, if >10 days of glucose and accelerometry data were captured, no redeployment took place.

To associate the glucose information captured by the glucose sensor, three measures of glucose variability were used within this study: mean daily glucose, SD of glucose, and mean amplitude of glucose excursions (MAGE). Mean glucose and SD of glucose were indicated as the most common and easily interpreted metrics [24], and MAGE is considered the gold standard for glucose variability measurement [25]. Glucose data were downloaded and processed using a semiautomated approach. The glucose reader logged data in two ways: (1) via automatic scans (every 15 min) and (2) via user manual scans (frequency dictated by participants). Only automatic scans were used within these analyses. If any data were missing, a manual adjustment using a user scan data point within ±3 min was made, or data were replaced using linear interpolation if below three adjacent data points were missing. Consecutive values ≥90% (86/96) for the day, including both waking and sleeping time, denoted a valid file and was carried forward for analysis. This decision was made to ensure that a true representation of glucose parameters was evaluated against PA. The largest block of data points was then analyzed using EasyGV software (University of Oxford).

### Statistical Analyses

For each participant, up to 13 complete days (cases) were available, as the first and last day were partial days. Days that did not meet the valid day criteria for accelerometry or glucose were deemed a nonvalid day overall. Only participants that had ≥7 coupled valid accelerometry, and glucose days were carried forward into the analyses.

Generalized estimating equations (GEEs) were used to estimate associations between sedentary time, light PA, and MVPA with mean glucose, SD of glucose, and MAGE. The correlation structure was evaluated through modeling changes in the quasi-likelihood under independence model criterion (QIC) value. Both unstructured (does not assume the magnitude of correlation between observations) and autoregressive (assumes a closer relationship between two observations taken closer together) correlation structures were assessed [26], with the autoregressive structure indicating a better fit for all models because of lower QIC values. Therefore, the autoregressive correlation structure was utilized for all analyses.

Models were calculated univariably and adjusted for age, sex, accelerometer wear time, and percentage body fat. This analysis extended further to investigate whether fitness-related differences existed for the associations between behavior and glycemic variability. Additional GEE analysis was conducted for those individuals who were deemed as having low fitness levels or had fitness levels within the needs improvement, fair, and good health benefit zones of the mCAFT. Individuals were placed within a fitness category based upon their aerobic fitness score that was derived using the O_2_ cost of the final stage they attained and their weight and age [27]. Wear time adjustment was included within the analyses to account for the variation in the amount of accelerometer wear time. Sensitivity analyses were also run without wear time for all models to investigate the influence of noncompliance on the associations. Although the valid day criterion for this study was ≥600 min, many participants had wear times well above this threshold.

Independent *t*-tests were also conducted to assess if participant characteristics differed between included and excluded participants. Finally, to investigate whether there were significant fitness-related differences in PA behaviors between low fitness and high fitness groups, analysis of covariance (ANCOVA) tests were conducted, adjusted for wear time.

## Results

### Participant Characteristics

Of the 84 individuals that expressed interest in the study, 76 were screened, and 36 were deemed ineligible to participate. Eight individuals were not screened as they initially expressed their interest but did not reply to our screening requests. Of those that were eligible, 5 withdrew before participating, leaving 35 participants who participated in the study. Six further individuals did not meet the activity and glucose coupled valid day criteria, and therefore, 29 were carried forward for analysis. The participants were aged 44.9 years (SD 9.1), had a mean BMI of 25.3 kg/m^2^(45%, 13/29 overweight, 14%, 4/29 obese), and predominantly self-reported themselves as white (90%, 26/29).

All participants had fasted blood glucose concentrations <7.0 mmol/L; however, 4 participants had fasted glucose concentrations suggesting prediabetes (ie, ≥5.6 mmol/L) [28]. Independent *t*-tests confirmed that there were no significant differences in the participant characteristics (demographics, anthropometrics, cardiometabolic risk factors, or fitness) between those who were included in the analysis and those who were excluded. Compared with a representative sample of Canadian adults that underwent the mCAFT, the average age matched (age range); VO_2_ max difference was 5.7 mg/kg/min greater for the whole sample and split by sex; both males (10.3 mg/kg/min) and females (2.5 mg/kg/min) had a greater average difference in VO_2_ max [29]. Participant characteristics are presented in Table 1.

### Device Compliance

In total, 6 participants had sensors prematurely removed because of sensor error (n=3), perceived discomfort wearing the sensor (n=1), or the adhesive failed before the 10-day threshold (n=2). Two participants out of the 6 participants did not receive a redeployment as sufficient data were collected. When participants failed to scan the glucose sensor within 8 hours, data were lost from the device, and the next available data point was the first available automatic scan, 8 hours before the latest scan. This often resulted in a temporal drift of the data, and for this reason, the amount of data points may have been 95 or 97, instead of 96 for a complete file (4 scans per hour). Fortunately, for the 29 participants over 13 days of complete wear, missing data represented only 2.70% (973/36,035) of total data points.

On average, each participant had 34 missing data points over the 13 days, equivalent to 8.5 hours. Two participants accounted for 43.17% of the missing data (420/973 data points), and removing their data reduces the average to 6.75 hours. Only 4 participants had no missing data out of the sample, and an additional 5 had below 2 hours of missing data. An outline of all glucose data processing information is presented in Table 2.

Accelerometry compliance was high with 83% (24/29) of the sample achieving 14 valid days (>600 min) of wear. When both the glucose data and the accelerometry data were overlaid to assess joint sensor compliance, the combined valid days was 11.6 (SD 1.5), which demonstrates an overall high level of device compliance.

### Associations Between Glycemic Variables and Behavior

Assumptions of linearity and normally distributed residuals were checked visually using residual and P-P plots, and multicollinearity was assessed using variance inflation factors (VIFs). VIF values were <2.7 in all models, which suggested no issues with multicollinearity.

Comparisons between movement behaviors (sedentary time, light activity, and MVPA) and glycemic variables (mean glucose, SD of glucose and MAGE) using GEE analysis for the whole sample is presented within Tables 3-5. GEE analysis revealed no significant associations between daily mean glucose, SD of glucose or MAGE with sedentary time, light activity, or MVPA for the whole sample.

An age and sex-adjusted health benefit zone was calculated for all participants using the mCAFT submaximal fitness test results. An analysis was calculated to ascertain the effect of only using participants categorized as having low fitness (needs improvement, fair, and good). GEE results for this lower fitness group are also presented in Tables 3-5. Univariable analyses revealed that light activity and MVPA were significantly associated with mean glucose for the low fitness group (*P*=.03; *P*=.001), with sedentary time also significant once wear minutes, age, sex, and percentage body fat were adjusted for (*P*<.001). SD of glucose was positively associated with sedentary time but inversely associated with light activity after adjustment in the low fitness group, with a similar trend occurring for the associations with MAGE.

**Figure 1:**
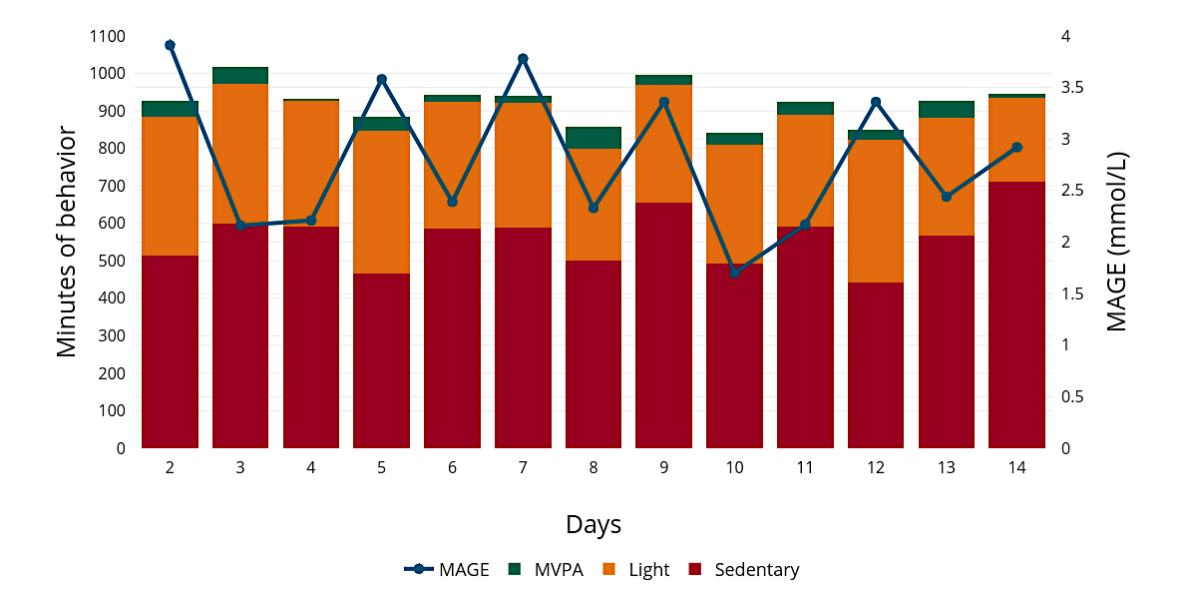
Minutes of sedentary, light, moderate to vigorous physical activity (MVPA) and mean amplitude of glycemic excursions (MAGE) over 13 days. Days 1 and 15 were half days and were omitted as there was insufficient data. The data represent a randomly selected female participant presenting low fitness.

The results of the sensitivity analyses produced comparable results for the adjusted models; however, sedentary time became nonsignificant without adjusting for accelerometer wear time for mean glucose and SD of glucose within the low fit models. No significant differences were observed for behavioral variables between low and high fitness groups (*P*>.05). Figure 1 represents daily summaries for behavior (sedentary, light, and MVPA) and MAGE for a typical participant who achieved 13 full valid accelerometer and glucose sensor days of wear.

**Table 1:** Characteristics of the study sample. Valid accelerometry ≥600 min; valid glucose ≥90% (86/96) of daily data points.

Characteristic			Mean (SD)	n (%)	Median	Interquartile range
**Demographics**						
	Age, years		44.9 (9.1)	—	44.0	15.0
	Sex, male		—	12 (41)	—	—
	Ethnicity, white		—	26 (90)	—	—
**Anthropometrics**						
	Body fat (%)		27.0 (9.7)	—	25.6	15.7
	**BMI^a^ (kg/m^2^)**		25.3 (4.1)	—	25.3	6.0
		Overweight	—	13 (45)	—	—
		Obese	—	4 (14)	—	—
	Waist circumference (cm)		85.0 (11.2)	—	82.9	13.2
**Cardiometabolic risk factors**						
	Mean systolic blood pressure (mmHg)		122.4 (11.7)	—	122.0	14.3
	Mean diastolic blood pressure (mmHg)		75.6 (7.0)	—	76.0	6.8
	Total cholesterol (mmol/L)		4.7 (0.8)	—	4.6	0.9
	Low-density lipoprotein cholesterol^b^ (mmol/L)		2.9 (0.5)	—	2.9	0.7
	High-density lipoprotein cholesterol (mmol/L)		1.5 (0.4)	—	1.4	0.6
	Triglycerides^b^ (mmol/L)		0.9 (0.2)	—	0.9	0.4
	Glucose (mmol/L)		4.9 (0.6)	—	4.9	0.7
	Glycated hemoglobin (%)		5.3 (0.4)	—	5.3	0.5
	Grip strength (combined kg)		71.9 (22.4)	—	69.5	36.0
	Quadriceps maximal voluntary contraction (nm)		139.7 (51.8)	—	132.0	66.0
**Fitness**						
	VO_2_ max^c^ (mL/kg/min)		41.4 (9.8)	—	39.0	11.2
	**Fitness score**		375.1 (84.3)	—	366.8	131.9
		Needs improvement	—	2 (7)	—	—
		Fair	—	10 (34)	—	—
		Good	—	4 (14)	—	—
		Very good	—	7 (24)	—	—
		Excellent	—	6 (21)	—	—
**Behavior**						
	Wear time per valid day (min)		895.1 (58.8)	—	883.8	85.4
	Sedentary time per valid day (min)		576.4 (67.8)	—	566.9	100.7
	Light physical activity per valid day (min)		269.1 (59.8)	—	271.1	86.3
	MVPA^d^ per valid day (min)		49.6 (29.9)	—	42.3	35.9
**Glucose**						
	Mean glucose (mmol/L)		5.1 (0.5)	—	5.0	0.8
	SD of glucose (mmol/L)		0.9 (0.2)	—	0.9	0.3
	Mean amplitude of glycemic excursions (mmol/L)		2.4 (0.6)	—	2.3	1.0
**Number of valid days**						
	**Valid accelerometry days**		13.8 (0.6)	—	14.0	0.0
		12	—	2 (7)	—	—
		13	—	3 (10)	—	—
		14	—	24 (83)	—	—
	**Valid glucose monitoring days (n)**		11.7 (1.5)	—	12.0	2.0
		7	—	1 (3)	—	—
		8	—	1 (3)	—	—
		10	—	2 (7)	—	—
		11	—	7 (24)	—	—
		12	—	8 (28)	—	—
		13	—	10 (35)	—	—
	**Overall valid combined analysis days (n)**		11.6 (1.5)	—	12.0	2.0
		7	—	1 (3)	—	—
		8	—	1 (3)	—	—
		10	—	2 (7)	—	—
		11	—	8 (28)	—	—
		12	—	8 (28)	—	—
		13	—	9 (31)	—	—

^a^BMI: body mass index.

^b^VO_2_ max=32.0 + (16.0 x VO_2_[L/min]) − [0.24 x Age] − [0.17 x weight (kg]) [21].

^c^n=28.

^d^MVPA: moderate to vigorous physical activity.

**Table 2:** Glucose data processing details. Due to the way the sensor was deployed, data processing information was calculated using full days only (days 2-13) and sensor information refers to the whole monitoring period. The maximum number of points per day was 96. Replaced values were taken from user scans if ±3 minutes; data points were interpolated if ≤2 adjacent values were missing; missing values represent data points not replaced or interpolated; if a sensor was not redeployed, data were treated as missing.

Data processing characteristics	n (%)
Average available data points per day	93 (97)
Average largest continuous block of data	91 (95)
Average number of valid days	12 (92)
Total data points replaced	28 (0.08)
Total data points interpolated	58 (0.16)
Total missing values not replaced or interpolated	973 (2.70)

**Table 3:** Associations between physical activity and mean glucose for the whole sample and for the low fitness group. Model one represents a univariable association, and model two is adjusted for wear minutes, age, sex, and percentage body fat.

Models		Mean glucose, beta (95% CI)	*P* value
**Model one**			
	Sedentary minutes all	0.00006 (−0.00018 to 0.00030)	.63
	Light minutes all	−0.00005 (−0.00040 to 0.00029)	.76
	MVPA^a^ minutes all	−0.00053 (−0.00128 to 0.00023)	.17
	Sedentary minutes low fit	0.00016 (−0.00011 to 0.00043)	.25
	Light minutes low fit	−*0.00042*^b^ (−0.00080 to *−0.00004*^b^)	*.03*^b^
	MVPA minutes low fit	−*0.00160*^b^ (*−0.00255*^b^ to *−0.00064*^b^)	*.001* ^b^
**Model two**			
	Sedentary minutes all	0.00019 (−0.00018 to 0.00055)	.32
	Light minutes all	−0.00005 (−0.00041 to 0.00031)	.77
	MVPA minutes all	−0.00053 (−0.00127 to 0.00022)	.17
	Sedentary minutes low fit	*0.00067*^b^ (*0.00030*^b^ to *0.00103*^b^)	*<.001* ^b^
	Light minutes low fit	−*0.00042*^b^ (*−0.00078*^b^ to *−0.00006*^b^)	*.02* ^b^
	MVPA minutes low fit	*−0.00154*^b^ (*−0.00250*^b^ to *−0.00058*^b^)	*.002* ^b^

^a^MVPA: moderate to physical activity.

^b^Significant results.

**Table 4:** Associations between physical activity and SD of glucose for the whole sample and for the low fitness group. Model one represents a univariable association, and model two is adjusted for wear minutes, age, sex, and percentage body fat.

Models		SD of glucose, beta (95% CI)	*P* value
**Model one**			
	Sedentary minutes all	0.00005 (−0.00021 to 0.00030)	.73
	Light minutes all	−0.00019 (−0.00058 to 0.00020)	.33
	MVPA^a^ minutes all	0.00008 (−0.00043 to 0.00059)	.76
	Sedentary minutes low fit	0.00017 (−0.00024 to 0.00057)	.42
	Light minutes low fit	−*0.00046*^b^ (*−0.00090*^b^ to *−0.00002*^b^)	*.04* ^b^
	MVPA minutes low fit	−0.00040 (−0.00146 to 0.00066)	.46
**Model two**			
	Sedentary minutes all	0.00018 (−0.00015 to 0.00051)	.29
	Light minutes all	−0.00025 (−0.00063 to 0.00012)	.19
	MVPA minutes all	0.00012 (−0.00036 to 0.00059)	.62
	Sedentary minutes low fit	*0.00059*^b^ (*0.00037*^b^ to *0.00081*^b^)	*<.001* ^b^
	Light minutes low fit	−*0.00062*^b^ (*−0.00085*^b^to *−0.00039*^b^)	*<.001* ^b^
	MVPA minutes low fit	−0.00044 (−0.00130 to 0.00041)	.31

^a^MVPA: moderate to physical activity.

^b^Significant results.

**Table 5:** Associations between physical activity and mean amplitude of glycemic excursions for the whole sample and for the low fitness group. Model one represents a univariable association, and model two is adjusted for wear minutes, age, sex, and percentage body fat.

Models		Mean amplitude of glycemic excursions, beta (95% CI)	*P* value
**Model one**			
	Sedentary minutes all	0.00035 (−0.00053 to 0.00123)	.44
	Light minutes all	−0.00079 (−0.00204 to 0.00046)	.22
	MVPA^a^ minutes all	−0.00008 (−0.00173 to 0.00157)	.93
	Sedentary minutes low fit	0.00074 (−0.00073 to 0.00221)	.33
	Light minutes low fit	−*0.00156*^b^ (*−0.00291*^b^ to *−0.00022*^b^)	*.02* ^b^
	MVPA minutes low fit	−0.00076 (−0.00492 to 0.00341)	.72
**Model two**			
	Sedentary minutes all	0.00088 (−0.00023 to 0.00199)	.12
	Light minutes all	−0.00107 (−0.00232 to 0.00018)	.09
	MVPA minutes all	0.00004 (−0.00155 to 0.00162)	.96
	Sedentary minutes low fit	*0.00202*^b^ (*0.00131*^b^*to 0.00273*^b^)	*<.001* ^b^
	Light minutes low fit	−*0.00216*^b^ (*−0.00285*^b^ to *−0.00146*^b^)	*<.001* ^b^
	MVPA minutes low fit	−0.00130 (−0.00429 to 0.00169)	.39

^a^MVPA: moderate to physical activity.

^b^Significant results.

## Discussion

### Principal Findings

Using accelerometer-measured PA and sedentary time, this study revealed an observable relationship between movement behaviors and glycemic variability, but only for those presenting lower fitness. The notion that lower fitness individuals can gain greater glycemic benefits from being more physically active is supported by previous experimental research that assessed glycemic responses after implementing light activity breaks [20]. In that particular study, individuals with lower cardiorespiratory fitness gained more favorable glucose responses compared with being sedentary, leading the authors to conclude that those with lower fitness levels may have the most to gain from replacing sedentary time with PA [20]. Additionally, when modeling the substitution of sedentary time for either light activity or MVPA, those with lower fitness (women <32 and men <35 ml/kg/min) and higher glucose (≥6.1 mmol/L) have been shown to benefit more than high fitness participants, even after adjustment for sex, age, educational level, smoking, and psychosocial stress [30]. These findings appear to support the hypothesis that although those with lower fitness may see daily differences in their glucose concentrations as a result of PA, those with higher fitness may keep their glucose concentrations within healthy ranges as a result of physiological adaptions, such as changes in insulin sensitivity [31,32].

Being physically active, especially after meals, is associated with a blunted glucose response as a result of the body using the supply of glucose already in the blood stream [33]. This may partly explain the observed inverse relationship between light activity and glucose variability in this study. Giving behavioral context to the magnitudes of the associations, if sedentary time is increased by 60 min, representing the duration of an average TV show, mean glucose could rise by 0.04 mmol/L for lower fitness individuals. Alternatively, increasing MVPA by 60 min per day could decrease mean glucose by 0.09 mmol/L, which is greater than the daily mean glucose fluctuations of 0.02 mmol/L of the low fit sample. Additionally, conducting 60 min of light activity could also decrease MAGE by 0.13 mmol/L, which represents 9% of average MAGE values (1.4 mmol/L) for a healthy white individual [34]. Over time, regularly engaging in PA may result in favorable changes to the glucose profile by blunting the glucose response or by initiating a faster return to normal levels (or euglycemia). Although sedentary time and light activity were associated with MAGE, no significant association was found with MVPA. Due to the 95% CIs being comparatively larger than the CIs for both sedentary and light activity, larger samples may be required to decrease the level of error for MVPA.

SD of glucose was associated with both sedentary time and light activity minutes in the low fitness group. Describing the spread around the mean, the average daily deviations were −0.0102 and −0.0001 mmol/L for both the low and high fit participants, respectively. Demonstrating a very low level of daily deviation in this study, SD of glucose may not be a viable marker of glycemic control within nondiabetics and/or populations presenting large fluctuations in glucose concentrations. Nevertheless, larger, more controlled samples would be needed to determine if this measure of glucose variability may be more beneficial in populations experiencing fluctuating glucose (higher SD of glucose); for example, individuals with prediabetes or type 2 diabetes.

It has been acknowledged that the relationship between behavior and glucose variability is complex and may differ depending on the amount of data captured, which is a product of sensor wear. Although the threshold for valid glucose data was set at ≥90% (86/96) of daily values, accelerometer wear time was set at ≥600 min in line with previous studies assessing habitual PA. Although most days comfortably exceeded the valid day threshold, there is some variation in the amount of wear. Indeed, the sensitivity analyses revealed that when wear time was removed from the low fitness models, the associations between sedentary time and mean glucose and SD of glucose became nonsignificant. Indicating that any difference in monitor wear largely influences the accrual of sedentary time, it is therefore important to investigate the influence of wear time when calculating physiological-behavioral models. Nevertheless, it is unknown whether reductions in wear are because of intentional device removal or extended periods of sleep duration. If the reason is the former, it would have important implications for the associations with acute health outcomes such as glucose.

For instance, Figure 1 from day 6 to day 7 illustrates a small difference in wear of 3 min but an increase in MAGE (day 6=2.39 mmol/L, day 7=3.78 mmol/L). It is hard to determine why the increase has been brought about in this instance and whether it is because of activity not captured by the accelerometer despite high wear across both days (day 6=944 min, day 7=941 min). Although wear time was adjusted for within the analyses, if it is imperative for individuals to wear the devices during *all* waking hours to show activity-related declines in glucose, then participant adherence may be challenging, given traditional wearable monitors can accrue steps intermittently, but still sum up to a goal at the end of the day. As a result, encouraging the deployment of 24-hour monitoring may help minimize the influence of missing data because of nonwear.

The data presented in this study provide evidence of the existence of an observable, acute stimulus-response model of increased PA that may yield measured changes in daily summaries of glycemic variability. If this information could be displayed in real time to users as actionable feedback, it may help support individuals to link together action (behavior) with the physiological consequence of their actions (health) [13]. Future research should focus on providing this feedback to users to observe how people respond (ie, do they change their behavior having seen their behavioral and physiological feedback?). However, scientists should not fall into the trap of more data being better by default; the information must be comprehended in a way that motivates action by the user that can be sustained [35].

Using glucose information in real time or at a bout level could further increase the potency of the feedback and also reduce the rate of temporal discounting. Although receiving real-time glucose feedback is potentially a richer feedback source than daily summaries, the physiological responses to movement behaviors are not necessarily routinely predictable. For instance, if a bout of PA is initiated, interrupted, and then recommenced, any change in glucose cannot be solely attributed to the first or second bout. This type of analysis would require highly complex computational algorithms, as the processing pipeline would need to consider, among others, the following issues:

- Behavior bout duration: what duration is considered a bout and can the bout be interrupted?
- Duration of the bout effect: how long does the increase or decrease in glucose last and can it be modified by behavior of a specific intensity?
- Sedentary time or PA: how does historical hourly, daily, or weekly movement behaviors influence the bout duration and effect?
- Glucose concentrations: how do historical glucose concentrations influence future concentrations?
- User characteristics: how do characteristics such as type 2 diabetes risk, fitness, sex, and age modify the associations?

Although there are clearly challenges to overcome, the innovative practice of providing people with daily actionable insights related to the physiological consequences of being more physically active (and/or less sedentary) may act as a potent driver for lifestyle behavior change. Scientists that have an interest within this area of research will be boosted by the new High-level global Commission on Noncommunicable Diseases that urges “...new approaches and action on a dramatically different scale if we are to stop people dying unnecessarily from noncommunicable diseases” [36]. These sentiments certainly resonate with the National Health Service Digital data and information strategy given its mission “…to empower the health and care system to be intelligent in the way it uses data and information to drive improvements in health and care, by delivering world class data and analytics services through the highest level of skills, expertise, tools, techniques and technology” [37].

### Limitations

This study is one of the first to investigate glycemic variability and PA behaviors using objective monitoring technologies in a free-living setting over an extended period of wear (13 complete days); however, there are a number of limitations that must be discussed.

The small number of participants within this study was chosen to balance feasibility and cost, although the participant pool could be considered homogenous (University location and ethnicity). Due to the lack of dietary information, which is a significant glucose input mechanism, the estimates should be interpreted cautiously given that dietary intake will likely have influenced magnitudes observed. Food diary information (pen and paper format) was collected for 4 days from second day of deployment. That said, the information was deemed unreliable because of the amount of missing food entries from the diaries and coding database. Self-reported dietary diaries have previously been called into question [38], and although the conclusions of Archer and Blair could be considered too far-reaching [39], alternative methods of food collection should be utilized in the future as it has suggested that self-reported methods should not be used for measures of energy intake [40].

The mCAFT was chosen because of its submaximal nature and the ease of the stepping modality and thus, its successful use in population public health research. That said, the gradings of the progressive stages are not as finely tuned as could be achieved when using a gas analysis system to quantify VO_2_ peak. Additionally, menstrual status was not captured, which has been shown to influence postprandial but not fasting concentrations of glucose [41]; therefore, menstruation would need to be adequately modeled in future studies to assess the implications for wearable glucose monitoring interventions, especially those that utilize the Freestyle Libre as interventions may span multiple weeks.

Sedentary time was measured using count-based accelerometry, which has the limitation of not detecting specific postural changes [42] and is instead measuring *stationary* behavior (lying, reclining, sitting, or standing with no ambulation) [43]. Similarly, the choice of cut-points can influence the data [44] and should therefore be taken into account when drawing conclusions. The valid day criterion for glucose data was chosen to be conservative but has not been validated. More work is required to ascertain what level of missing data is acceptable and does not introduce an unacceptable level of variability.

This study faced glucose sensor deployment issues and missing data. Coupled with the invasiveness and recurring costs of glucose monitoring, there are a number of hurdles that researchers need to be aware of when using such devices in research. Additionally, as one of the first studies utilizing physiological feedback, we are unable to determine whether the information given back to the participants had any influence on the direction of glucose and/or movement behaviors. Although most continuous glucose monitoring validation studies (including the Freestyle Libre) have been predominately conducted on individuals diagnosed with diabetes, studies conducted in small samples of Japanese adults suggest that the Freestyle Libre may overestimate glucose levels in response to an isolated meal load [45,46]. Nevertheless, as all measurements from one study fell within zones A and B of the Parkes error grid, further investigation using larger samples are required to confirm the extent of the overestimation. Further analysis and alternative glucose monitoring technologies and future iterations of devices could perhaps ameliorate these hurdles.

### Conclusions

This study demonstrates that there are both positive and inverse associations between accelerometry-derived behavioral and glycemic variability within a small sample of white, middle-aged, low fitness adults living without a clinical diagnosis of diabetes. Future research should expand on these findings within populations who may exhibit greater variability. Investigations should also be conducted to ascertain if bout-related information can be extracted, to assess whether this data could be used to educate and influence lifestyle behaviors.

## Abbreviations

ANCOVA: analysis of covariance
BMI: body mass index
CPM: counts per minute
GEE: generalized estimating equations
MAGE: mean amplitude of glycemic excursions
mCAFT: modified Canadian Aerobic Fitness Test
MVPA: moderate to vigorous physical activity
PA: physical activity
QIC: quasi-likelihood under independence model criterion
SIGNAL: Sensing Interstitial Glucose to Nudge Active Lifestyles
VIF: variance inflation factor
